# Acoustic Monitoring Confirms Significant Poaching Pressure of European Turtle Doves (*Streptopelia turtur*) during Spring Migration across the Ionian Islands, Greece

**DOI:** 10.3390/ani13040687

**Published:** 2023-02-16

**Authors:** Christos Astaras, Zoi-Antigoni Sideri-Manoka, Manolia Vougioukalou, Despina Migli, Ioakim Vasiliadis, Sotirios Sidiropoulos, Christos Barboutis, Aris Manolopoulos, Michalis Vafeiadis, Savas Kazantzidis

**Affiliations:** 1 Forest Research Institute, ELGO-DIMITRA, Vasilika, TK 57006 Thessaloniki, Greece; 2 WildTrack, Durham, NC 27708, USA; 3 Hellenic Ornithological Society, TK 10437 Athens, Greece; 4 School of Biology, Aristotle University of Thessaloniki, TL 54124 Thessaloniki, Greece; 5 Department of Earth Sciences, Uppsala University, SE-75236 Uppsala, Sweden

**Keywords:** illegal killing of birds, wildlife crime, passive acoustic monitoring, Afro-Palearctic migrant, Ionian Islands, spring poaching

## Abstract

**Simple Summary:**

The European turtle dove (*Streptopelia turtur*) is a migratory species that overwinters in sub-Saharan Africa, migrating to Europe each spring to breed. Over the past four decades, turtle dove populations have declined by as much as 79%, making the species vulnerable to extinction. A major threat to the species is illegal killing (poaching) during its spring migration through the Mediterranean coasts of Europe. According to the international action plan for the conservation of the species, eradicating this threat is key for reversing the European turtle dove population declines by 2028. In this study, we used a network of acoustic sensors to record the gun hunting pressure at known hunting sites in the Ionian Islands, Greece—a known poaching hot-spot—over four spring migrations (2019–2022). Based on the number of gunshots recorded, we estimate that up to 57,095 turtle doves were killed or injured across the region in 2021. We anticipate that these findings will generate the resolve needed among responsible authorities to eradicate the spring migration in the Ionian Islands, and propose the roll out acoustic monitoring grids in additional poaching hot-spots along the migration routes of the turtle dove.

**Abstract:**

The European turtle dove (*Streptopelia turtur*) is an Afro-Palearctic migrant whose populations have declined by 79% from 1980 to 2014. In 2018, the International Single Species Action Plan for the Turtle Dove (ISSAP) was developed with the goal of enabling, by 2028, an increase in turtle dove numbers along each of the three migration flyways (western, central, eastern). To achieve this, the illegal killing of turtle doves, a critical threat to the species, has to be eradicated. The Ionian Islands off the west coast of Greece lie on the eastern flyway and are considered a major turtle dove poaching hot-spot during spring migration. Quantifying wildlife crime, however, is challenging. In the absence of a reliable protocol for monitoring spring poaching levels, the agencies tasked with tackling the problem have no means of assessing the effectiveness of the anti-poaching measures and adapting them if required. Using passive acoustic monitoring (PAM) methods, we recorded gun hunting intensity at known turtle dove poaching sites during the 2019–2022 spring migrations (2–10 sites/season) with unprecedented spatial and temporal resolution. Based on published gunshot to killed/injured bird ratio for similar species (corroborated with discussions with local hunters) and an estimate of the proportion of hunting sites monitored by our PAM grid (using gunshot detection range estimates from control gunshots), we estimated that in 2021, up to 57,095 turtle doves were killed or injured across five Ionian Islands (Zakynthos, Paxi, Antipaxi, Othoni, and Mathraki). The 2022 estimate was almost half, but it is unclear as to whether the change is due to a decline in poachers or turtle doves. We propose ways of improving confidence in future estimates, and call for a temporary moratorium of autumn turtle dove hunting in Greece—as per ISSAP recommendation—until spring poaching is eradicated and the eastern flyway population shows signs of a full recovery. Finally, we hope our findings will pave the way for the development of PAM grids at turtle dove poaching hot-spots across all migration flyways, contributing to the global conservation of the species.

## 1. Introduction

There are over 120 Afro-Palearctic migrant bird species which fly northwards each spring to breed in Europe, returning to sub-Saharan Africa to overwinter [1]. An assessment of 38 of them from 1980 to 2009 by the Pan-European Common Bird Monitoring Scheme (PECBMS) showed 71% having a declining population trend that was often more pronounced than that of the resident or short-distance migrants [2]. Due to the migrant species’ reliance during their annual cycle on resources extending across multiple ranges of countries, they are exposed to a diverse array of anthropogenic pressures, including habitat degradation due to land-use, and climate change and direct mortality, including over-harvesting [3].

In part due to the predictable timing of the migration of entire populations twice each year, migrant species are especially vulnerable to illegal killing or trapping at sites across the Mediterranean where the birds stop-over to rest and/or refuel along their main migratory routes [4]. Recognizing the conservation importance of this threat, the Convention on the Conservation of Migratory Species of Wild Animals adopted in 2014 a resolution and established a task force to address bird crime in the region (”Intergovernmental Task Force to address illegal killing, taking and trade of migratory birds in the Mediterranean” [5]).

The European turtle dove (*Streptopelia turtur*) (hereafter turtle dove) is an Afro-Palearctic migrant which has experienced a 79% population decline from 1980 to 2014 [6]. As a result, it was uplisted as Vulnerable on the IUCN Red List in 2015 [7] and in 2018 an International Single Species Action Plan for the Turtle Dove (hereafter ISSAP) was developed [8]. The turtle doves migrate along three flyways: the western over Iberia, the central over Malta and Italy, and the eastern over Greece, Bulgaria and Turkey [9]. The ISSAP aims to set the ground for an increase in the species’ numbers along each flyway by 2028, and eventually a favorable population status and the downlisting of the species to Least Concern in the IUCN Red List [7,8].

To achieve these objectives, first the current population decline of turtle doves must be halted. The ISSAP identifies illegal killing as a one of the critical threats that turtle doves face that needs to be eradicated in the European Union (EU) and reduced in other range countries. Evaluating the scale of illegal killings is an essential first step towards this goal according to the ISSAP, along with enhancing the enforcement of hunting regulations especially at hot-spots of illegal turtle dove killings [8].

The Ionian Islands of western Greece are one such hot-spot [4]. Spring migration hunting is strictly forbidden by EU legislation as it removes from the breeding stock individuals of the highest fitness (i.e., survivors of post-breeding migration and overwintering), affecting the population recruitment process [10]. Despite the hunting ban an estimated 69,000 turtle doves are illegally killed across the Ionian Islands each year during the species’ spring migration [11], which takes place from late March to late May [7,10]. However, quantification of wildlife crime, such as poaching, is complex [11,12] and verification of illegal harvest numbers difficult, which is why the number of turtle doves killed in the Ionian Islands has been disputed [8].

Passive acoustic monitoring (PAM) has gained increased recognition in recent years as an efficient method for estimating with high spatial and temporal resolution gun hunting pressure across large areas [13,14]. Typically, a grid of semi-autonomous acoustic sensors are deployed across a landscape to record continuously the ambient sound (soundscape) for extended periods of time (weeks or months), with the data subsequently being scanned either manually or by using detection algorithms to locate the events of interest (i.e., the time and date of gunshots per sensor). Compared to other hunting/poaching estimation methods, PAM does not suffer from possible misleading reporting or bias sampling of human subjects (e.g., as can be the case with questionnaire surveys [15,16]), or relying on difficult to interpret indices of hunting (e.g., the detection of cartridges, hunting posts, etc. [13]). Crucially, the gun hunting estimates are readily verifiable by all interested parties via examination of the raw acoustic data [13].

Recognizing the importance for the European turtle dove’s conservation to quantify the scale of the spring migration poaching pressure across the Ionian Islands—a hot-spot for illegal killing of the species along its eastern migration flyway, we recorded over four spring migrations the gun hunting activity at historically important turtle-dove poaching sites in the Ionian Islands using passive acoustic monitoring methods (PAM). The goal is to provide the robust field evidence needed to assess existing and adapt future strategies for the eradication of this wildlife crime in the region—as per the Objective 2 of the International Single Species Action Plan for the Turtle Dove (ISSAP). Finally, it is our desire that the present study will pave the way for the adoption of similar PAM methods at turtle dove illegal killing hot-spots across all migration flyways and the enforcement of wildlife laws contributing to the global conservation of the species.

## 2. Materials and Methods

### 2.1. Study Area

**Figure 1:**
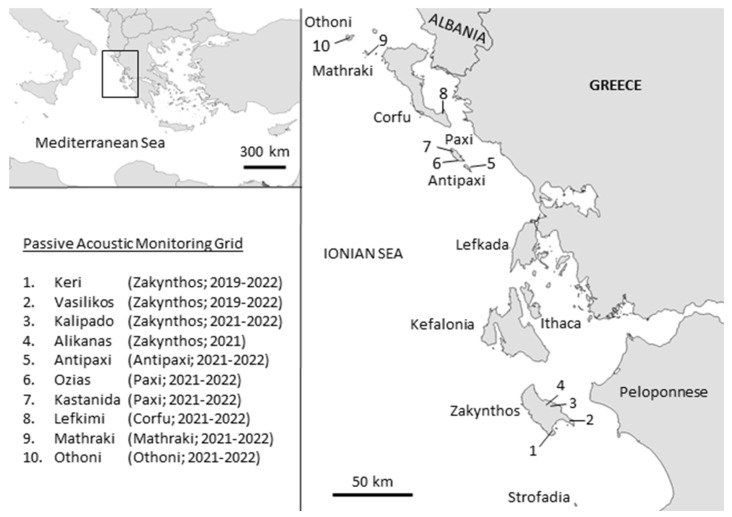
Location, name, and years of operation of the passive acoustic monitoring grid sensors in the Ionian Islands.

The Ionian Islands (2608 km^2^) are a group of Greek islands stretching across a north south axis in the Ionian Sea, parallel to the western coast of the country (Figure 1). There are seven principal islands (north to south: Corfu, Paxi, Lefkada, Ithaki, Kefalonia, Zakynthos, Kythera) and 22 smaller islands. The human population in 2011 was 208,000, of which 85% are in Corfu, Zakynthos and Kefalonia, while 12 smaller islands are uninhabited.

The vegetation of coastal areas is characterized by wild olive (*Olea europaea*) and carob trees (*Ceratonia siliqua*) [17], as well as large swaths of cultivated olive groves. As elevation increases, forested areas are dominated by kermes oak (*Quercus coccifera*), turkey oak (*Quercus cerris*), hop hornbeam (*Ostrya carpinifolia*), and—eventually—Greek fir trees (*Abies cephalonica*) [18]. The rich biodiversity of the Ionian Islands is protected via two National Parks, 19 Natura 2000 Sites (Sites of Community Importance and Special Protection Areas), and 11 Wildlife Refuges [10]. The island complex lies along the eastern migration flyway of Afro-Palearctic migrant bird species, including the European turtle dove.

Culturally, despite the vicinity to mainland Greece, the Ionian Islanders maintain and are proud of a distinct cultural identity with its own linguistic idiom, cuisine, architecture, songs and customs—in part a result of three centuries of Venetian (and briefly French and British) rule, before joining Greece in 1864.

The local hunters consider the poaching of turtle doves during spring migration to be such a local custom and continue the illegal hunts despite it being banned in Greece since 1985. Originally, the main motivation for poaching turtle doves was self-consumption and gifting surplus birds to friends and family, but it has progressively turned into a mean of demonstrating wealth and social status [10]. Typically, groups of hunters use established hunting spots (“posta”) located along known coastal landing sites of turtle doves or within olive orchards further inland (pers. obs.). Such “posta”, which are often within private properties, are rented for considerable fees to poachers during the regular turtle dove migration period [10]—which in the Ionian Islands is from April to May (peak at the end of April). The turtle doves briefly rest to refuel before continuing their migration to their breeding grounds in mainland Europe.

The islands of Zakynthos, Corfu, Paxi, Antipaxi, Othoni and Mathraki were historically hot-spots of spring turtle dove poaching, which is why they became this study’s focus for estimating spring poaching activity using passive acoustic monitoring methods (PAM).

### 2.2. Data Collection

The study commenced with the pilot deployment of two acoustic sensors at the Keri and Vasilikos sites of Zakynthos Island from 23 April to 31 May 2019 (Figure 1). The collected data were used to (a) confirm the feasibility of monitoring gun hunting activity in the region using PAM methods, (b) assess the performance (recall rate) of the algorithm used to detect gunshots, and (c) refine the recording schedule in the following years. Since very few gunshots occurred between 10 pm and 7 am (summer time), recording around the clock was deemed unnecessary in subsequent years.

Strict nationwide travel restrictions (“lockdown”) related to the COVID-19 pandemic meant that in spring 2020 only the two Zakynthos sites could be monitored once again; this time for the entire migration season (15 March to 31 May; 7 am to 10 pm). During the 2021 and 2022 spring migrations, a ten sensor acoustic grid was deployed to record gun hunting activity during the same period (15 March to 31 May; 7 am to 10 pm), across six Ionian Islands (Zakynthos n = 4—including the Keri and Vasilikos sites of 2019 and 2020, Paxi n = 2, Antipaxi n = 1, Corfu n = 1, Othoni n = 1, and Mathraki n = 1).

The islands were selected based on prior knowledge of where spring poaching of turtle doves has been most pronounced in the past. The sites where the acoustic sensors were placed within these islands were selected based on ground surveys for “posta” (identifiable from semi-permanent hides and old spent cartridges) and discussions with local hunters. An exception was the sensor placed at the Lefkimi wetland in Corfu, where the aim was to determine whether poaching occurred there, rather than estimating the levels of known poaching activity. In Zakynthos, two sensors (Keri and Vasilikos) were selected to represent poaching pressure at the two southern peninsulas, where the turtle doves make landfall, whereas the other two (Alikanas and Kalipado) were selected to represent poaching pressure inland, where the turtle doves spread out across a landscape mosaic of villages, hedges, and olive groves. In the case of the smaller islands of Paxi, Antipaxi, Othoni, and Mathraki, the sensors were placed at sites that would provide a good coverage of the islands’ countryside.

All sensors (SWIFT rugged version, Cornell University, Ithaca, NY, USA) were placed at approximately 1.8 m on tree trunks or branches, using foliage to reduce exposure to wind and passerby detection. Areas with high background noise (e.g., frequent car traffic, stream water flow) were avoided, when possible. The sensors were powered by 12 D alkaline batteries and were scheduled to record continuously from 7 am to 10 pm (15 h) at 8 KHz sampling rate and 33 dB gain, recording the data on an SD card (Class 10). The SWIFT sensor microphone is omni-directional and has a signal to noise ratio of >58 dB (PUI Audio/Part Number: POW-1644L-B-LW100-R).

In order to determine the range at which gunshots were detectable by our sensors, in February 2022 we conducted control gunshots at known distances from three SWIFT sensors placed in line at 100 m intervals (Figure S1, Table S1 for details regarding the field design). The sensors recorded using the same settings (sampling rate, gain) as those deployed in the Ionian Islands. We used a shotgun (Benelli Montefeltro) and cartridges (Kirgias—Navaro 32, cal. 12, tipo 2, 12/70 mm, 32 g) typical of those used for bird hunting in Greece. At 15 locations (70 m to 1100 m from the closest of the three sensors), two gunshots were fired at 90 degrees angle to the sensors (i.e., the shots were not fired towards or away from the sensors). During the first gunshot, the gun had a slight downward tilt, simulating shooting at ground quarry 30 m away. During the second gunshot, the gun was aimed upwards with approximately 45% angle, simulating a shot at flying quarry. The control gunshots were all taken within an hour during a cloudless winter day with little to no wind (0–2 m/s). The terrain was level, with little vegetation (plowed fields). Background noise was low (33–37 db; 2 min average; decibel meter PeakTech 8005).

In 2021, the Department of Wildlife and Game Management (Hellenic Ministry of Environment and Energy) requested that the Forest Services of Zakynthos and Corfu took increased measures to combat illegal spring hunting of migratory birds in the islands Zakynthos, Paxi, Antipaxi, Corfu, Mathraki, and Othoni from 20 April to 20 May. Specifically, anti-poaching patrols were to be conducted by forest rangers, game guards and park rangers (with the logistical support of the police and coast guard, if required). We obtained from the relevant authorities the reports of these patrols (time, date, location, findings, arrests) in order to examine, using the data from the acoustic sensors, and their effectiveness in curbing poaching pressure.

### 2.3. Data Analysis

The sound files (.wav format) from each acoustic sensor were scanned using a gunshot data template detector (DTD 1.5.6) developed by Cornell University [19]. A purpose-trained data analyst visually and acoustically reviewed the putative gunshots with a threshold score >0.4 using Raven Pro 1.5 (Cornell University) (Sony Professional MDR-7506 headphones; spectrogram settings: window size 2048, contrast 35, brightness 50). The 0.4 threshold was selected based on a preliminary analysis of a subset of data, as it reduced false positives—and hence the review time—while maintaining a high recall rate (“sensitivity”; true positives/(true positives + false negatives) [20]). The recall rate of the gunshot detection algorithm was calculated by manually detecting all gunshots in a subset of days (known to have at least some gun hunting activity) representing all sensors and years. We used a strict protocol when verifying and counting the gunshots of a selection window (sound clip), ensuring that gunshots detected ad hoc while reviewing the data were not counted, to ensure reliable comparisons of poaching activity across the sites and years (Figure S2). Each year, a single analyst reviewed the data, who was trained and supervised by CA.

To estimate the number of turtle doves killed across the Ionian Islands, we (a) adjusted the recorded gunshots by the recall rate, (b) assumed all gunshots were for turtle doves, (c) used a 1:5 kill rate (i.e., five shots to kill a bird), and (d) estimated the proportion of each island’s hunting sites (“posta”) monitored by the acoustic grid based on field observations, discussions with locals, and the gunshot detection range estimated from the control gunshots. The turtle dove is effectively the only species poached using guns during spring migration in the Ionian Islands [10]; smaller migratory bird species are caught with lime sticks in Greek islands [21]. In addition, all spring hunting is banned in Greece (hunting season: end of August—mid-February, depending on quarry). Therefore, the assumption that the detected gunshots represent turtle dove poaching is a safe one to make. Regarding the kill rate used, a similar rate has been reported for mourning doves (*Zenaida macroura*) in the USA, based on the evaluation of 5094 gunshots fired by 53 hunters [22]. If injured birds were to be added, the kill/injure rate of that study increased to 1:3. Injury to a bird such as a turtle dove that is depleted from crossing the Sahara desert and the Mediterranean Sea is likely to lead to mortality. Discussions with three anonymous local hunters suggest that the 1:5 killed is a conservative estimate.

In order to roughly determine the destination breeding populations of the turtle doves migrating through the Ionian Islands during spring, we used data on turtle dove ring recoveries provided by the Hellenic Bird Ringing Center. Since recent evidence suggests the turtle doves using the central/eastern routes follow a clockwise loop migration (i.e., the autumn route lies east of the spring migration route) [9], we used only the recovery data of turtle doves that were either (a) ringed in Greece during spring and recovered elsewhere during the breeding season, or (b) ringed during the breeding season and recovered during spring in Greece. Then, we drew a minimum convex polygon containing the ring recoveries, as per Hahn et al. [23], and considered all of the countries that the polygon spans as the breeding grounds of turtle doves migrating through our study area. Estimates of turtle dove population sizes and trends in these countries, as reported in the ISSAP [8], were used to discuss the potential impact of the Ionian Islands’ spring poaching pressure to them.

## 3. Results

### 3.1. Poaching Intensity and Patterns

All sensors recorded for the same number of days each year (2019: 39 days, 585 h/sensor; 2020–2022: 77 days, 1155 h/sensor). A total of 54,014 gunshots were detected in the 2019–2022 spring migration acoustic data (a mean of 1.38 gunshots/sound selection window; a range of 1–12 gunshots) (Table 1) using the gunshot algorithm. Most gunshots were recorded at the Keri and Vasilikos sites, located at the southern coast of Zakynthos island (81% of all years; 71% and 67% of the 2021 and 2022 data, respectively). No poaching activity was recorded at the Lefkimi site, Corfu Island, in either 2021 or 2022.

**Figure 2:**
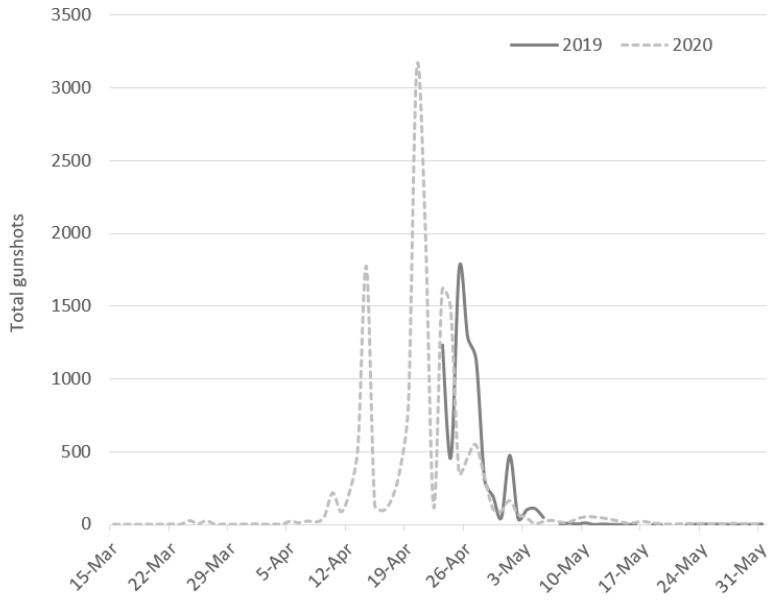
Daily variation in total poaching activity at the Keri and Vasilikos (Zakynthos Island) sites during the 2019 (23 April to 31 May) and 2020 (15 March to 31 May) spring migrations.

**Figure 3:**
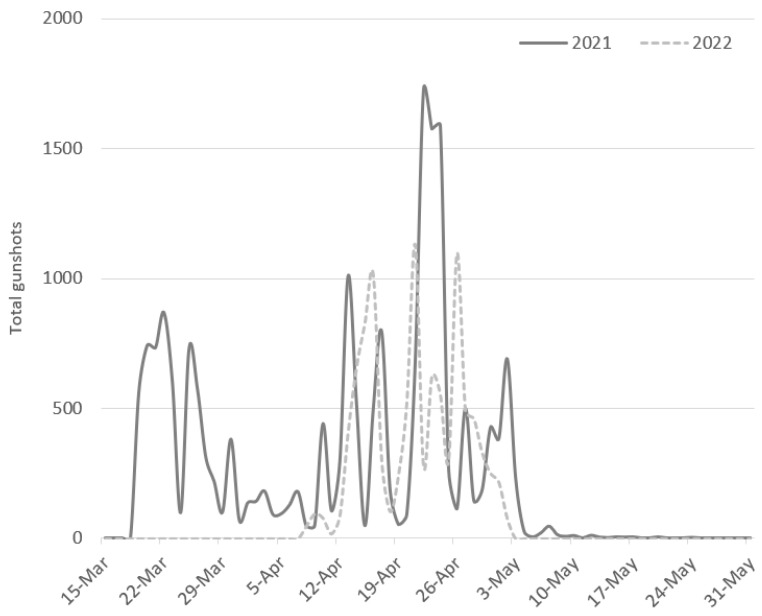
Daily variation in the total poaching activity at nine sites during the 2021 and 2022 (15 March to 31 May) spring migrations. Data from the Kalipado (Zakynthos) acoustic sensor are not included for the sake of comparison, as that sensor malfunctioned in 2022. Almost all the gunshots in March were recorded at the Vasilikos (Zakynthos) site.

Across the years, the poaching was most intense (86% of gunshots) between 10 April and 5 May, after which gunshots sharply declined to near zero (Figure 2 and Figure 3). An exception was the Vasilikos site in 2021, when 78% (n = 5732) of the gunshots at that site occurred from 15–30 March. Only in Antipaxi were gunshots recorded in that same period (n = 157; 7% of the site’s 2021 gunshots). Regardless of the sensor, month, or year, poaching activity showed sharp peaks and troughs, even within a period of a few days (Figure 2 and Figure 3).

**Figure 4:**
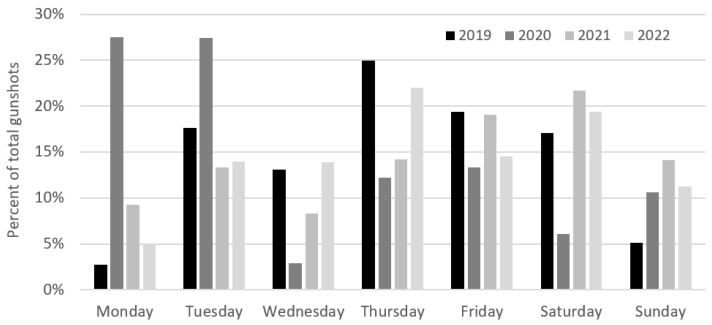
Weekly poaching pattern during the 2019–2022 spring migrations (n = 54,014).

**Figure 5:**
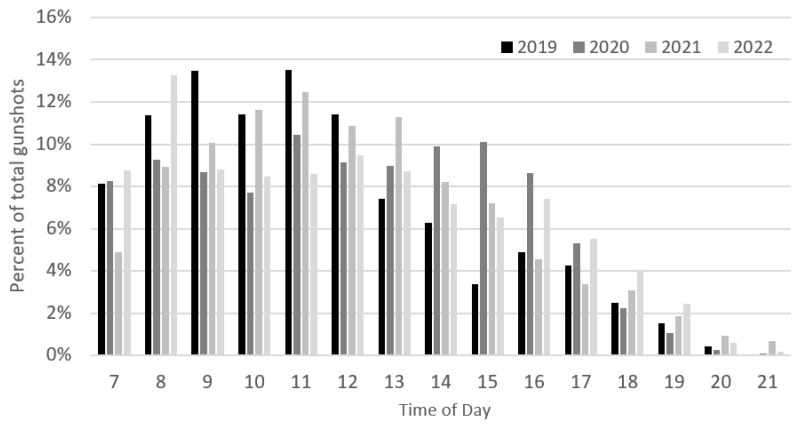
Diel poaching pattern during the 2019–2022 spring migrations (n = 54,014). Data were only recorded from 7 am to 10 pm.

There was no inter-annual pattern regarding poaching activity and the day of the week (Figure 4). In terms of the time of the day, poaching typically commenced soon after 7 am, increased after 8 am, and remained high until noon, declining sharply after 5 pm (>93% gunshots between 7 am and 5 pm, across all years), (Figure 5).

Based on the gun hunting activity at Keri and Vasilikos, which were monitored over all four years (2019: 23 April–31 May; 2020–2022: 15 March–31 May), there is evidence of a 10% decline in poaching pressure from 2020 to 2021, followed by an additional 49% decline from 2021 to 2022 (Table 1). A similar, or higher, 2022 decline in detected gunshots was observed in the other sensors, with the exception of Alikanas, Zakynthos, which showed an 83% increase compared to 2021. Since the Kalipado, Zakyntos sensor malfunctioned in 2022, it was not possible to determine whether the Alikanas 2022 increase represented a broader trend in poaching pressure at the inland posta of Zakynthos.

### 3.2. Assessment of the Detection Algorithm

A manual review of 15 days of acoustic data (representing all sensors 2019–2021) located 6816 gunshots. The gunshot algorithm, using the 0.4 score threshold, correctly detected—i.e., had a recall rate of—62.4% of these gunshots (annual range 55.4–64.1%). In effect, for every 100 gunshots detected, it is safe to assume that there were an additional 60.3 gunshots missed (false negatives). Therefore, the total gun hunting pressure should be adjusted by a factor of 1.603.

**Figure 6:**
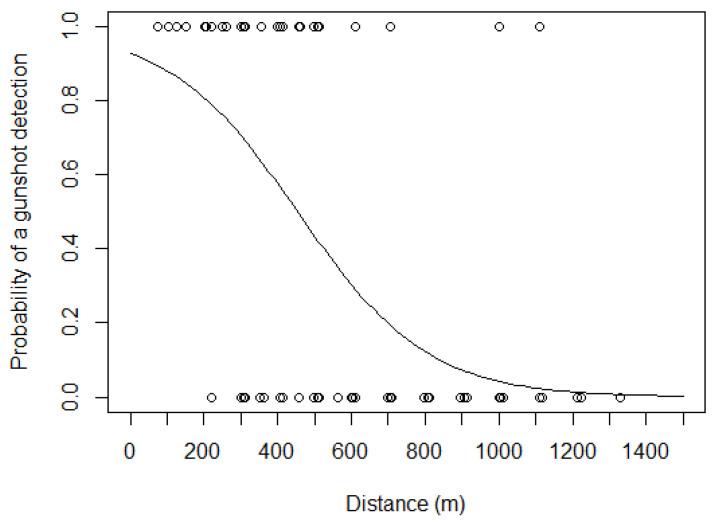
Fitted logistic regression of the probability of a gunshot being detected using the DTD 1.5.6 gunshot detection algorithm with threshold 0.4, plotted as a function of the acoustic sensor’s (SWIFT rugged model) distance to the gunshot.

A manual review of the control gunshots detected both acoustically and visually all gunshots, up to a distance of 1100 m (n = 87). The gunshot detection algorithm located 36 of those (41.4%) (Figure 6). Therefore, the poaching activity reported at each monitored site reflects—at most—an area of 3.8 km^2^.

### 3.3. Number of Illegally Killed Birds

Adjusting the total gun hunting pressure by the detection algorithm’s recall rate, we estimate a total of 33,511 gunshots to have occurred within the acoustic grid in 2021—the year when all 10 sensors operated (Table 2). Based on field surveys, discussions with locals, the consideration of topography, and a review of land-cover/land-use maps and aerial photographs, we estimate that these gunshots reflect the poaching activity at 25%, 30%, 50%, and 50% of the Paxi, Antipaxi, Othoni, and Mathraki posta, respectively. In Zakynthos, we estimate that the Keri and Vasilikos sensors captured the poaching of 20% of the posta located along the southern coast, where turtle dove “flotillas” (as the migrating flocks are locally known) are hunted as they make landfall. The Kalipado and Alikanas sensors, we estimate, capture the poaching pressure of 10% of the inland posta, where the poaching pressure is lower as the birds move across a wider front through the island’s countryside. Based on the above, and on a kill rate of 1:5, during the 2021 spring migration, a total of 34,257 turtle doves were killed across the five Ionian Islands (Table 2). This number increases to 57,095 if we include injured birds as well, using a 1:3 kill/injure rate.

Based on 19 ring recoveries, turtle doves migrating through Greece and the Ionian Islands during spring head to the breeding grounds of 13 countries, ranging from Greece in the south to Poland in the north (Figure S3). The estimated combined turtle dove population for these countries is 763,250 breeding pairs according to conservative estimates (Table S2). For the populations where a population trend is known (n = 9), four are decreasing, four are stable, and one is fluctuating (Table S2).

### 3.4. Anti-Poaching Patrol Effectiveness

In 2021, the Zakynthos and Corfu Forest Services conducted a total of 48 (9 April to 15 May; 15 locations) and 52 (24 April to 20 May; 9 locations) patrols, respectively, specifically aimed at reducing the illegal poaching of birds during spring migration. With the exception of 10 patrols by the Corfu based forest guards, who reported “isolated gunshots at a long distance”; none of the patrols reported any other evidence of poaching, and no arrests, further investigations, or confiscations of killed birds and/or hunting equipment were made.

In the data available to us, the area of a patrol was identifiable only by a toponym (often the name of a nearby village). Seventeen of the patrols occurred at sites monitored by the passive acoustic monitoring grid; the data analysis later confirmed poaching activity during the 2021 spring migration (Zakynthos n = 12; Othoni = 3; Mathraki = 2) (Table S3). An additional 19 patrols included the Lefkimi, Corfu site, where we did not record any gunshots in the acoustic data (either in 2021 or 2022). The Paxi and Antipaxi islands were not patrolled at all, while the patrols at Othoni and Mathraki occurred too late in the season (after 4 May) to detect any of the poaching activity that had peaked and faded out >5 days earlier. Similarly, the patrols at Alikanas (n = 1) and Vasilikos (n = 3) occurred too early or too late in the season. Concurrent anti-poaching patrols with post hoc acoustically confirmed gun hunting activity occurred only during seven patrols at the Keri (n = 5) and Kalipado (n = 2) sites. Data limitations did not allow for a statistical analysis on the possible effects of the patrols on the poaching activity during these sites and dates, and we do not have information on the exact routes of the patrols in the given areas. However, the Keri patrols were well timed, with 1108 gunshots being detected in the acoustic data during the patrols’ dates (mean 411 ± 204 SD gunshots/day; range 8–578; values not adjusted for the detection algorithm’s recall rate). Still, the patrols did not report any evidence of poaching activity.

## 4. Discussion

Quantifying the spring migration poaching pressure of the European turtle dove (*Streptopelia turtur*) is key for assessing and adapting existing strategies for eradicating this critical threat to the species. Only then can the observed declining population trends in the three migration flyways (western, central, and eastern) be reversed by 2028—a primary objective of the International Single Species Action Plan for the Turtle Dove (ISSAP) [8]. Our study provides verifiable, quantitative evidence that the Ionian Islands of Greece remain a poaching hot-spot for turtle doves along the eastern flyway, depriving each year the species’ pre-breeding stock of tens of thousands of birds of the highest fitness.

Even though the ring recovery data provide only a rough description of the breeding populations impacted upon by the Ionian Islands’ spring poaching, the illegal turtle dove harvest that we reported in 2021 exceeded 3.7% of the combined breeding pairs of these 13 countries—and it was larger than the entire breeding population of four of them (Table S2). Incurring such losses at only a fraction of the turtle dove’s eastern spring migration flyway no doubt hinders, or possibly even cancels, the conservation measures taken at these countries to reverse the existing decreasing population trends.

The findings also give credence to earlier reported estimates on the scale of the spring poaching pressure in the region (i.e., 69,000 turtle doves/year [11]). Our reported annual illegal harvest may be lower, but it does not include some Ionian Islands (e.g., Kefalonia, most of Corfu) or known poaching areas on the western coast of the Peloponnese. Regardless, the passive acoustic monitoring (PAM) data presented here serve as a baseline of the current status quo, enabling the relevant authorities to assess with unprecedented spatial and temporal resolution the effectiveness of future conservation measures. The striking difference between the PAM and the patrol-based findings on the level of poaching activity in 2021 highlights the urgency and importance of reconsidering the current anti-poaching strategy. Clearly, when a patrol fails to report any poaching activity in the Keri peninsula (12 km^2^), Zakynthos, on a day that our acoustic sensor—located meters off a main, public forest road—recorded 578 gunshots (927 adjusted for the detector’s recall rate; 13 April 2021; Table S2), there are issues beyond human and financial resource limitations that have to be addressed. The high 2020 and 2021 poaching pressure recorded by the PAM grid, at a time that strictly enforced COVID-2019 pandemic-related travel restrictions (”lock down”) were in place, confirms the held belief that most poachers reside locally. This complicates the task of the locally based patrol personnel, who live within these small island communities.

Nevertheless, there is the precedence of successfully eliminating turtle dove poaching at a previous hot-spot in the Ionian Islands—the two Strofadia islets (2.5 km^2^ and 0.1 km^2^) located 45 km southwest of Zakynthos (Figure 1), where spring poaching was rampant until the turn of the 21st century. Poaching ceased after the combined and sustained efforts of park/game guards, and police and coast guard forces, and the personnel of the Holy Metropolis of Zakynthos, to which the Strofadia islets belong. There are lessons from this success case that can be transferred to the rest of the Ionian Islands, starting with the will and commitment for change.

Converting gunshots to an estimate of illegal turtle dove harvests required the use of a gunshot to injured/killed bird ratio, and an estimation of the proportion of hunting sites surveyed by the PAM grid in each island. Improving our confidence in the values used would increase the accuracy and precision of current and future estimates. Based on discussions with local hunters (who wish to remain anonymous), the kill rate used is probably on the conservative side. While qualitative data akin to those used to calculate the kill rate of mourning doves (*Zenaida macroura*) [22] could be collected, we believe that resources should be best invested in improving the estimation of the percent of posta (hunting sites) that are acoustically monitored each year. This could be achieved by expanding the PAM grid, ensuring a better representation of the spring migration poaching areas, and the intra- and inter-annual poaching variations at them that our data showed. Given the decreasing costs of acoustic sensors (currently ~450 Euro/sensor, including import taxes for a SWIFT sensor) and the low operation cost (<Euro 8/month for batteries), doubling or tripling the PAM grid is within the budget range of wildlife conservation projects in the region. In addition, a citizen science project could be developed to collect information on the activity (or equally important inactivity) of known “posta”, at a coarser temporal scale. Such a project would have the additional benefit of engaging the public (e.g., conservation sensitive citizens, tourists, and local hunting club/Hellenic Hunting Confederation members), empowering them to be part of the solution of a local conservation challenge.

A challenge of the PAM data interpretation is that it is difficult to determine the cause of observed spatiotemporal changes in poaching pressure. For example, was the observed poaching decrease in 2022 compared to 2021 due to fewer poachers or fewer turtle doves passing through the monitored sites? A frequent synchrony in hunting peaks across sensors and the near universal poaching decrease in 2022 would suggest that poachers respond quickly to turtle dove availability, and therefore, the poaching decrease reflects fewer turtle doves that year. However, given the illegal nature of spring hunting, we do not have information on the numbers of poachers, and as such, we cannot convert gunshots to hunting opportunities per hunter—an abundance index that has previously been reported for monitoring the population trends of turtle doves during the autumn migration in Greece [24]. Direct counts of turtle doves in the Ionian Islands during spring migration are complicated by the fact the species’ migration movements occur both during the day and night [9,25], and weather conditions (especially temperature and wind) at the wintering and staging/stopover sites can affect the arrival times of migratory species [26,27]), which probably explains the early passage (second half of March) of a large wave of turtle doves through the Vasilikos peninsula in 2021. We propose that a turtle dove monitoring grid is developed along the PAM grid in the Ionian Islands, ideally equipped with avian radar systems able to detect approaching or overflying bird flocks, even at night [28,29]. Additionally, studies tracking the migration routes of turtle doves passing through the Ionian Islands and western Greece should be conducted, in order to identify the breeding populations that are affected the most by the spring poaching.

The provided information, in conjunction with the PAM estimates of the turtle doves killed, would be used to estimate the percent of the pre-breeding population off-take each year. Such knowledge can inform the population parameters used in the sustainable harvest models developed by the ISSAP [8].

## 5. Conclusions

To date, the Greek government has not approved the International Single Species Action Plan for the Turtle Dove, because it objects to the inclusion of measure 3.1.1, which calls for the implementation of a temporary moratorium of turtle doves, regardless of season, until an adaptive harvest management model framework is developed [8]. We believe that the evidence presented in this study regarding the persistence and scale of the spring migration poaching of turtle doves in the Ionian Islands should be seen as a call for reconsidering turtle dove conservation and management actions in Greece. With sustained political support for the strict enforcement of wildlife laws across the Ionian Islands, we believe that the spring poaching can be eliminated in less than five years. During this period, the passive acoustic monitoring grid should be maintained (and ideally expanded) to enable the annual assessment of the anti-poaching measures’ effectiveness. In addition, given that the spring poaching poses a threat to an already declining population of a species, we propose that a temporary autumn hunting moratorium is introduced in Greece until the eastern flyway population shows signs of a full recovery.

Finally, we propose the adoption of similar passive acoustic monitoring grids for estimating turtle dove illegal harvests across EU and non-EU spring migration poaching hot-spots, where the primary method of poaching is gun hunting. The data from such a broad network of monitoring grids could help to assess the progress towards the ISSAP’s goal of reversing, by 2028, the current population decline trends observed across the turtle dove’s migration flyways.

## Figures and Tables

**Table 1:** Number of verified gunshots per spring migration and site, and percentage change of poaching activity from 2021 to 2022.

Island	Site Name	2019	2020	2021	2022	2021–2022 % Change
Zakynthos	Keri (coast)	3603 *	6735	6651	3647	−45.2%
	Vasilikos (coast)	3687 *	8845	7361	3223	−56.2%
	Alikanas (inland)	-	-	919	1680	+82.8%
	Kalipado (inland)	-	-	1196	-	-
Paxi	Kastanida	-	-	1092	408	−62.6%
	Ozias	-	-	384	188	−51.0%
Mathraki	Mathraki	-	-	711	2	−99.7%
Othoni	Othoni	-	-	240	37	−84.6%
Antipaxi	Antipaxi	-	-	2351	1054	−55.2%
Corfu	Lefkimi	-	-	0	0	-
	Total	7290 *	15,580	20,905	10,239	−48.0% **

* Pilot year: Gunshots recorded only from 23 April to 31 May (vs. 15 March to 31 May in other years); ** Value estimated based on the total gunshots of all sensors, except Lefkimi, Corfu (no gunshots), and Kalipado, Zakynthos, which malfunctioned in 2022.

**Table 2:** Estimation of the number of turtle doves illegally killed during the 2021 spring migration across the Ionian Islands, based on (a) the total number of gunshots detected by the passive acoustic monitoring grid (10 sensors), (b) the adjustment of these gunshots, given the 62.4% recall rate of the gunshot detection algorithm, (c) the conversion of gunshots to killed birds using a 1:5 kill and a 1:3 killed or injured rate, and (d) extrapolation to the monitored Ionian Islands based on educated estimates on the probable proportion of the “posta” (hunting sites) monitored by the acoustic grid within each island.

Island	Gunshots Detected	Adjusted Gunshots for Recall Rate	Estimated Killed Turtle Doves (1:5 kill–1:3 Injure/Kill Rate) within the Monitored Sites	Estimated % Posta Surveyed	Estimated Killed–Killed/Injured Turtle Doves in the Ionian Islands
Zakynthos (south coast)	14,012	22,461	4492–7487	20%	22,461–37,435
Zakynthos (inland)	2115	3390	678–1130	10%	6781–11,301
Antipaxi	2351	3769	754–1256	30%	2512–4187
Paxi	1476	2366	473–789	25%	1893–3155
Othoni	240	385	77–128	50%	154–256
Mathraki	711	1140	228–380	50%	456–760
TOTAL	20,905	33,511	6702–11,170	-	34,257–57,095

## Data Availability

Data may be available from the authors upon request.

## Supplementary Materials

The following supporting information can be downloaded at: https://www.mdpi.com/article/10.3390/ani13040687/s1, Table S1: Distance matrix of the locations of the control gunshots (n = 15/2 shots fired at each location, for a total of 30 gunshots) and the three stations with Cornell Swift sensors; Table S2: Breeding population size (pairs) and short-term trend of overall populations of destiny countries for turtle doves passing through Greece during spring migration, as reported by Fisher et al. (2018); Table S3: Timing of anti-poaching patrols (dates marked by black squares) by the Forest Services of Zakynthos and Corfu during the 2021 spring migration; Figure S1: Field design of control gunshots; Figure S2: Description of protocol used to count gunshots (using Raven Pro 1.6) from the putative gunshot results (selection tables) produced by the DTD 1.5.6 gunshot detection algorithm; Figure S3: Minimum Convex Polygon containing all retrieved turtle doves ringed in Greece (n = 19) during spring migration.
