# Supplementary material for: Acoustic Monitoring Confirms Significant Poaching Pressure of European Turtle Doves (Streptopelia turtur) during Spring Migration across the Ionian Islands, Greece
Source: Animals (Basel). 2023 Feb 16;13(4):687. doi: 10.3390/ani13040687 (PMC9952176; doi:10.3390/ani13040687)

Supplementay materials:

Table S1: Distance matrix of the locations of the control gunshots (n=15 / 2 shots fired at each location, for a total of 30 gunshots) and the three stations with Cornell Swift sensors.

**Gunshot**	**Sensor 1 (m)**	**Sensor 2 (m)**	**Sensor 3 (m)**
GS01	1,116	1,224	1,329
GS02	1,001	1,109	1,214
GS03	897	1,005	1,110
GS04	798	906	1,011
GS05	700	808	913
GS06	600	707	812
GS07	497	604	708
GS08	400	507	611
GS09	350	457	562
GS10	301	408	512
GS11	249	356	460
GS12	202	310	414
GS13	151	259	363
GS14	102	205	308
GS15	74	127	220

Table S2: Breeding population size (pairs) and short-term trend of overall populations of destiny countries for turtle doves passing through Greece during spring migration, as reported by Fisher et al. 2018.

	Breeding Population (pairs)			
Country	Average	Min	Max	Population Trend
Austria	9,250	7,500	11,000	decreasing
Bosnia and Herzegovina	10,000	5,000	15,000	unknown
Bulgaria	67,500	35,000	100,000	unknown
Croatia	75,000	50,000	100,000	unknown
Czech Republic	75,000	50,000	100,000	moderate decrease
Greece	55,000	30,000	80,000	stable
Hungary	107,000	64,000	150,000	stable
Kosovo	9,000	7,000	11,000	unknown
N. Macedonia	40,000	20,000	60,000	stable
Poland	37,000	25,000	49,000	decreasing
Romania	21,0000	120,000	300,000	fluctuating
Serbia	46,000	39,000	53,000	decreasing
Slovakia	22,500	15,000	30,000	stable

Table S3: Timing of anti-poaching patrols (dates marked by black squares) by the Forest Department of Zakynthos and the Forest Department of Corfu during the 2021 spring migration.

Red bars and numbers show the intensity of the gun hunting activity at each site on a given date (gunshots detected using threshold 0.4 of the DTD 1.5.6 gunshot detection algorithm; results are not adjusted by the algorithm's recall rate0.

		Forest Department - Zakynthos				Forest Department - Corfu				
Month	Date	Vasilikos (Zante)	Keri (Zante)	Kalipado (Zante)	Alikanas (Zante)	Antipaxi	Kastanida (Paxi)	Ozias (Paxi)	Othonoi	Mathraki
MARCH	19/03/2021	560	0	0	0	0	0	0	0	1
	20/03/2021	742	0	0	0	0	0	0	0	0
	21/03/2021	718	0	0	0	5	0	0	11	0
	22/03/2021	866	0	0	2	0	0	0	0	0
	23/03/2021	603	1	0	0	0	0	0	0	0
	24/03/2021	91	7	0	0	0	0	0	0	0
	25/03/2021	729	0	0	0	0	0	0	0	0
	26/03/2021	529	0	0	0	45	0	0	0	0
	27/03/2021	311	0	0	0	0	0	0	0	0
	28/03/2021	217	0	0	0	0	0	0	0	0
	29/03/2021	94	6	0	0	0	0	0	0	0
	30/03/2021	272	1	0	0	107	0	0	0	0
	31/03/2021	62	4	0	0	2	0	0	0	0
APRIL	01/04/2021	122	8	0	5	0	0	0	0	0
	02/04/2021	127	9	0	5	0	0	0	0	0
	03/04/2021	179	1	0	0	0	1	0	0	0
	04/04/2021	80	2	0	0	0	1	7	1	0
	05/04/2021	93	0	0	0	0	0	0	0	0
	06/04/2021	125	1	0	1	0	0	0	0	0
	07/04/2021	141	17	0	0	1	0	0	19	0
	08/04/2021	46	0	2	0	0	0	0	0	0
	09/04/2021	39	5	3	3	0	0	0	0	0
	10/04/2021	383	50	1	5	1	0	0	0	1
	11/04/2021	19	82	3	2	0	1	0	1	0
	12/04/2021	95	172	4	3	14	0	0	0	8
	13/04/2021	18	578	7	21	331	20	6	1	35
	14/04/2021	2	448	9	9	56	0	2	0	0
	15/04/2021	6	9	2	13	19	0	1	0	0
	16/04/2021	20	414	23	28	26	3	4	3	0
	17/04/2021	5	445	8	18	124	90	51	55	7
	18/04/2021	5	121	51	40	10	0	0	6	4
	19/04/2021	5	8	17	4	18	3	0	5	9
	20/04/2021	13	28	18	7	24	2	2	1	13
	21/04/2021	13	264	20	20	270	29	0	25	51
	22/04/2021	1	978	43	104	271	129	59	10	174
	23/04/2021	3	784	50	33	258	242	97	32	127
	24/04/2021	1	931	11	13	277	286	7	44	33
	25/04/2021	6	158	21	38	41	4	3	0	13
	26/04/2021	2	59	29	21	1	1	0	13	16
	27/04/2021	3	221	12	33	86	33	13	1	109
	28/04/2021	1	41	6	3	32	16	13	7	32
	29/04/2021	3	112	23	42	4	7	0	4	13
	30/04/2021	2	310	34	45	47	20	1	0	1
MAY	01/05/2021	1	96	35	51	76	76	48	0	35
	02/05/2021	1	136	589	250	115	122	54	0	11
	03/05/2021	1	106	128	44	66	5	8	0	0
	04/05/2021	2	5	10	10	4	1	4	0	0
	05/05/2021	1	1	8	1	0	0	0	0	1
	06/05/2021	1	1	5	15	1	0	0	1	0
	07/05/2021	2	5	4	24	14	0	0	0	0
	08/05/2021	0	4	4	3	5	0	0	0	0
	09/05/2021	0	3	1	3	0	0	0	0	0
	10/05/2021	0	8	2	0	0	0	0	0	1
	12/05/2021	0	0	0	0	0	0	0	0	10
	13/05/2021	0	0	2	0	0	0	0	0	3
	14/05/2021	0	0	1	0	0	0	0	0	1
	15/05/2021	0	4	8	0	0	0	0	0	0
	16/05/2021	0	3	1	0	0	0	0	0	0
	17/05/2021	0	4	0	0	0	0	0	0	0
	20/05/2021	0	0	1	0	0	0	4	0	0
	24/05/2021	0	0	0	0	0	0	0	0	2

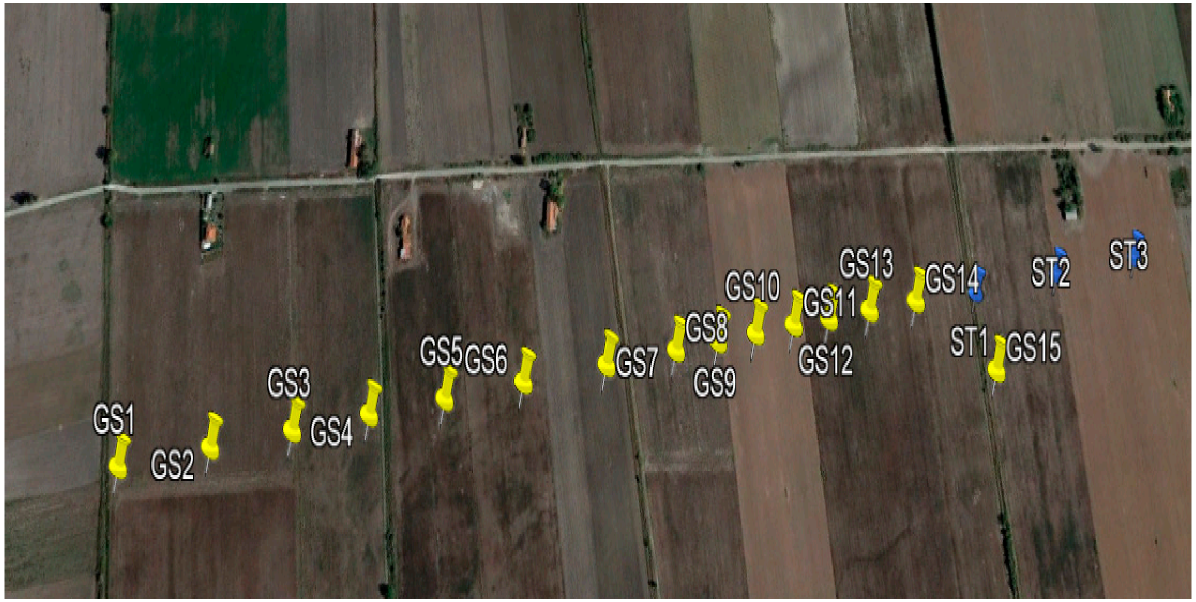

Figure S1: Field design of control gunshots

GS1 to GS15 (yellow pins) mark the 15 locations at which gunshots were fired, and ST1 to ST3 (blue pins) mark the acoustic stations where the SWIFT sensors were placed. See Table S1 for distance matrix of all gunshot locations and the three stations. [Scale: Blue pins are located exactly 100m apart]

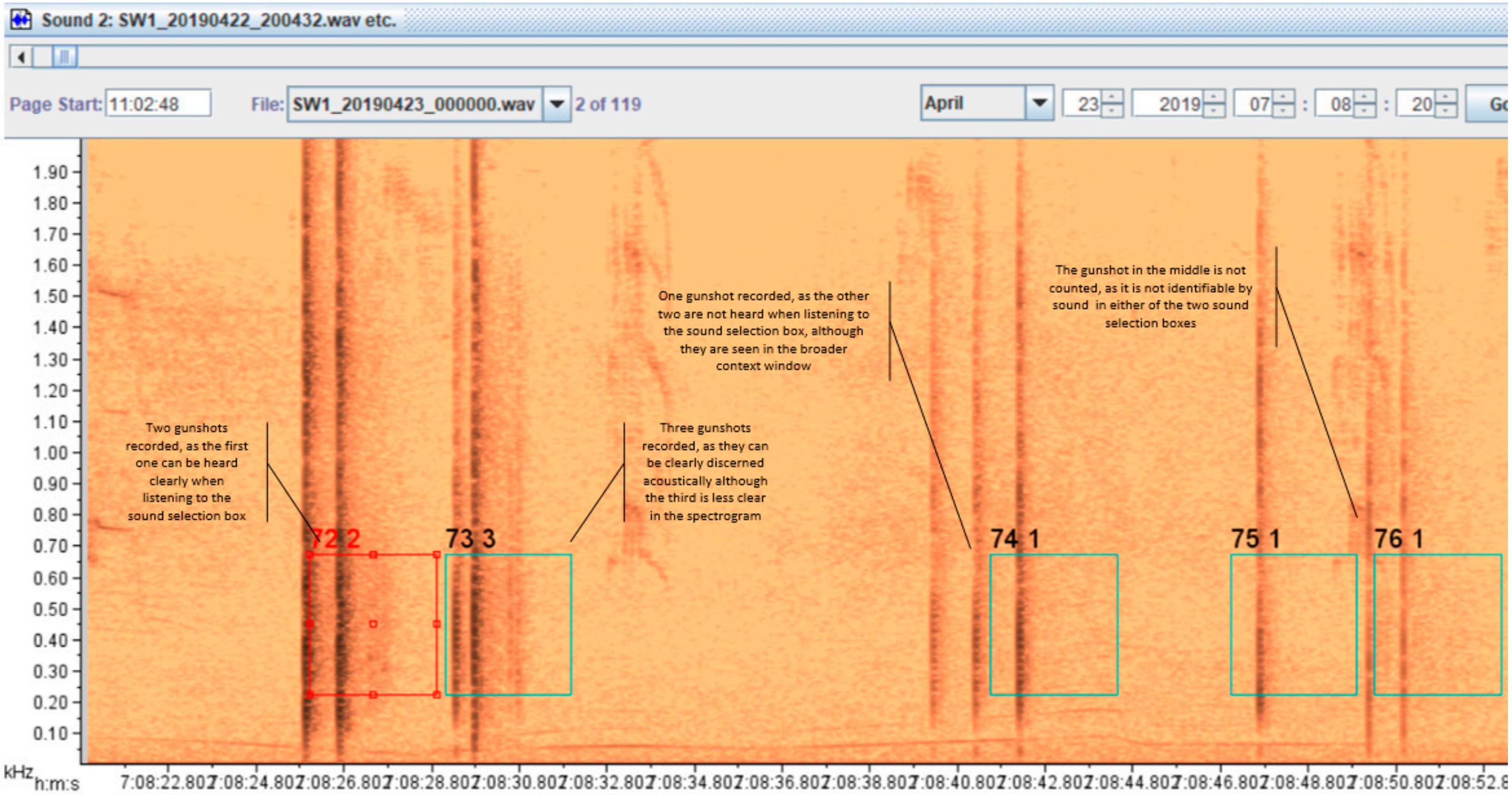

Figure S2: Description of protocol used to count gunshots (using Raven Pro 1.6) from the putative gunshot results (selection tables) produced by the DTD 1.5.6 gunshot detection algorithm. The figure shows as an example a 30 second spectrogram viewed in Raven Pro's context window.

Although at first glance one can count ten gunshots, only eight of those are contained within the four sound selection boxes (Nr 72 to 76) flagged by the detection algorithm for review. Acoustic review of the four sound selection windows detected one extra gunshot in box 73 (three in total, rather than two most easily seen), and confirmed that both gunshots can be heard in box 72 as although only the tail of the first one is heard, and can be identified as such with confidence. On the contrary, two gunshots right before boxes 74 and 76 are not counted as they can not be heard by listening to the sound selection box.

Figure S3: Minimum Convex Polygon containing all retrieved trurtle doves ringed in Greece (n=19) during spring migration. Populations span 13 countries (see Table S2)

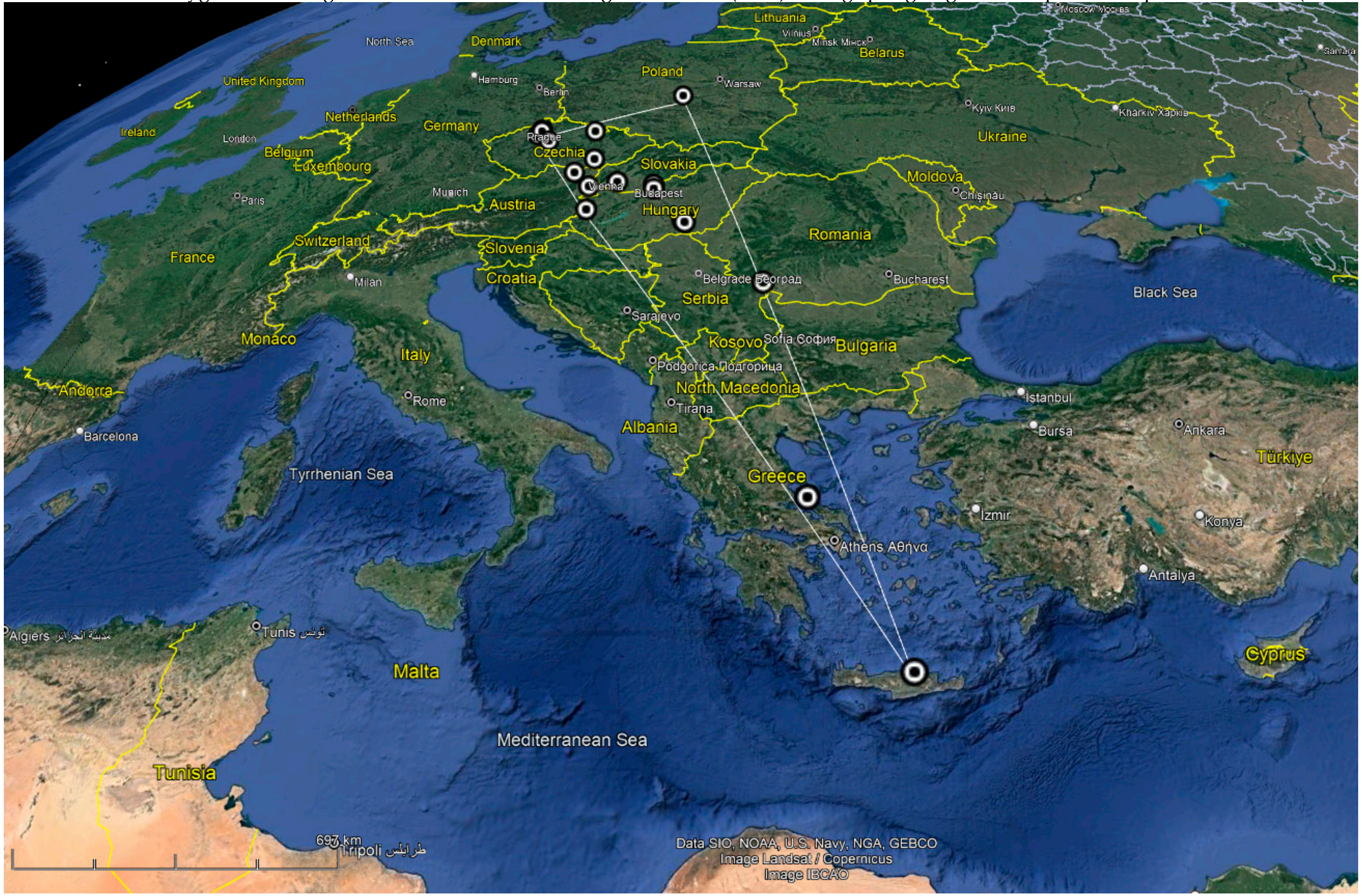

Supplement: Supplementary file 1 [file animals-13-00687-s001.zip › animals-2192002-SI.pdf]
